# Hemodialysis and Water Management in a Dialysis Unit in Morocco, an Approach to Dealing With Water Scarcity

**DOI:** 10.1111/hdi.13241

**Published:** 2025-04-01

**Authors:** I. Haddiya, I. Melhaoui, A. El Khalifi, S. Ramdani, Y. Bentata, F. Z. Berkchi

**Affiliations:** ^1^ Department of Nephrology Mohammed VI University Hospital Oujda Morocco; ^2^ Laboratory of Epidemiology, Clinical Research and Public Health, Faculty of Medicine and Pharmacy of Oujda University Mohammed First Oujda Morocco; ^3^ Hassan II Center of Hemodialysis and Chronic Diseases Oujda Morocco

**Keywords:** environmental impact, green dialysis, hemodialysis, sustainability, water management

## Abstract

**Introduction:**

Chronic kidney disease is a global public health issue, affecting approximately 10% of the world's population, and more than 3 million people living with kidney failure who are estimated to be on maintenance dialysis programs, with the majority receiving hemodialysis (HD). This treatment is particularly water‐intensive, posing a considerable challenge in regions experiencing water scarcity, such as Morocco.

**Methods:**

Our HD center in Oujda, Eastern Morocco, has implemented several key strategies to address water scarcity and ensure uninterrupted HD procedures during periods of hydric stress.

**Results:**

These strategies include expanding water storage capacities to safeguard against shortages, upgrading infrastructure to enhance water efficiency, and employing innovative technology for real‐time monitoring and management of water resources. Additionally, we collaborate closely with local water authorities to secure reliable water supplies and explore possibilities for water regeneration and recycling.

**Discussion:**

The rising demand for clean water, coupled with its increasing scarcity, presents a significant challenge for healthcare systems, particularly in the context of HD. Therefore, innovative approaches are essential to mitigate this issue. The concept of green dialysis, which focuses on reducing water usage and minimizing environmental impact, is emerging as a promising solution with measurable benefits. Implementing water‐efficient reverse osmosis systems has resulted in significant reductions in water waste, while real‐time monitoring and early warning systems have enhanced water security and operational efficiency. Additionally, initiatives exploring the reuse of reject water hold potential for further conservation. These tangible outcomes demonstrate how green dialysis practices can contribute to sustainable HD treatment, ensuring uninterrupted patient care, reduced resource consumption, and improved environmental stewardship in water‐scarce regions.

## 1 Introduction

Climate change is reshaping global water distribution, intensifying water scarcity, and threatening essential services, including healthcare. The WHO Alliance for Transformative Action on Climate and Health (ATACH) underscores the urgent need for health systems worldwide to enhance resilience against climate change‐related disruptions. ATACH promotes integrating climate adaptation into health policies, emphasizing sustainable resource use, including water security in medical facilities, to safeguard patient care in vulnerable regions [1]. The 2024 Lancet Countdown report highlights that health systems remain largely unprepared for climate‐induced challenges, with inadequate strategies to mitigate the impact of extreme weather events, water shortages, and resource depletion on healthcare delivery. The report stresses that urgent systemic adaptations are needed to ensure healthcare resilience, particularly in regions facing severe environmental stressors such as Morocco [2]. Many regions, including Morocco, are experiencing declining groundwater levels and freshwater shortages due to erratic precipitation, prolonged droughts, and increasing water demand from agriculture and industry [3, 4, 5]. These challenges place water‐dependent medical treatments, such as hemodialysis (HD), at significant risk, necessitating strategies to ensure a stable and adequate water supply for continued care delivery.

Morocco and the surrounding region face escalating water shortages, particularly in eastern areas where access to drinking water is limited. Healthcare services, including dialysis centers, are increasingly affected by this crisis, as HD requires substantial volumes of high‐purity water per session [6, 7, 8, 9]. The reliance of kidney replacement therapies on consistent water availability underscores the urgent need for resilience in kidney care delivery. While previous literature has extensively explored water conservation strategies in HD, less attention has been given to adaptation measures ensuring dialysis continuity during periods of water scarcity [10]. Given that agriculture alone accounts for 89% of Morocco's annual water consumption, healthcare facilities must not only adopt water conservation strategies but also develop resilient adaptation plans to safeguard patient care in times of water scarcity [8].

Our paper examines the state of water resources in Morocco and the national strategies in place to ensure a sustainable and adequate water supply. While previous literature has extensively explored methods to reduce water consumption in HD, less attention has been given to the adaptation challenges faced by dialysis centers when access to water becomes uncertain. Here, we highlight practical adaptation strategies that can be implemented in HD centers, particularly in regions prone to water insecurity. Our focus is on measures deployed at our HD center in Oujda, detailing how facilities can ensure dialysis continuity during water shortages through infrastructure adjustments, emergency water supply planning, and policy integration within national water management initiatives [6, 7, 8, 9].

### 1.1 National Water Management Initiatives

Morocco has made significant progress in the field of water management, thanks to its decades‐long policy of building dams, the hydro‐agricultural infrastructure built since independence, and proactive public policies aimed at making access to drinking water more widespread. Nevertheless, the country is not resting on its laurels and is continuing its momentum with recent royal initiatives led by the Ministry of Logistics and Water, mainly by creating the National Drinking Water Supply and Irrigation Program 2020–2027 [11, 12].

The program includes structuring projects in the water sector, particularly by mobilizing conventional and unconventional water resources to ensure resilience in water‐dependent industries, including healthcare [12]. Water demand management measures, such as network efficiency improvements and leak repairs, have already demonstrated their effectiveness in freeing up significant water volumes [13]. However, healthcare facilities, including dialysis centers, must integrate into these frameworks to ensure uninterrupted treatment in periods of extreme water scarcity.

Another main pillar of the program is the reuse of wastewater via the National Mutualized Liquid Sanitation Plan (PNAM) created in 2018. The annual volumes of wastewater discharges have risen sharply over the past decades, and went from 48 million m^3^ in 1960–700 million m^3^ in 2010 and are expected to continue to rapidly grow up to 900 million m^3^ in 2030 [14]. The PNAM aims to promote the reuse of treated wastewater to mobilize 474 million m^3^/year by 2030, used mainly for agricultural irrigation and watering golf courses and green spaces [15].

Lastly, Morocco has been implementing a national seawater desalination strategy, leveraging its 3500 km coastline. By 2030, the country aims to produce nearly 1.3 billion cubic meters of desalinated water annually [16]. While primarily intended for domestic, industrial, and agricultural use, desalination presents a potential adaptation pathway for healthcare facilities if integrated into emergency water supply planning.

### 1.2 Implications for HD Centers

As water scarcity intensifies, dialysis centers must move beyond traditional water‐saving measures and establish adaptive frameworks to secure alternative water sources, optimize supply chain resilience, and develop emergency protocols. Collaborating with national water agencies, integrating dialysis centers into broader water management policies, and investing in on‐site water storage or filtration solutions will be crucial for ensuring continued access to life‐saving HD treatment in the face of increasing water insecurity [17, 18].

## 2 Methods

The Hassan II HD center is located in Oujda, covering an area of over 500 m^2^ on two floors, and has 60 dialysis machines serving over 120 people who rely on dialysis therapies each day.

The establishment also includes a chronic kidney disease prevention unit with a young diabetic area, an adult area with consultation rooms, blood sampling, nutritional education, and a laboratory, as well as reception and rest facilities for people who live with kidney failure.

Currently, there is no peritoneal dialysis (PD) program at the Hassan II Dialysis Center. However, within the region, PD services are available at the Mohammed VI University Hospital Center. This highlights the regional disparity in dialysis modalities and the reliance on HD as the primary renal replacement therapy in Oujda.

The center roughly consumes 1,036,800 L (103 m^2^) of drinking water per day to ensure safe HD for its patients.

The reported 80,000 L of annual water use equates to approximately 500 L per dialysis session for a standard 4‐h treatment, three times per week regimen. However, previous estimates of 120–160 L per session referred only to the volume of water converted into dialysate, excluding wastage from reverse osmosis (RO) rejection and other system losses. The total water footprint per run is higher due to pretreatment losses, purification inefficiencies, and machine‐specific factors.

This variation highlights the importance of distinguishing between direct dialysate consumption and total facility water use. While efforts to optimize water efficiency—such as improving RO recovery rates and potential reject water reuse—have helped reduce overall waste, precise calculations depend on specific machine settings and operational protocols. Future analyses should aim to refine these estimates by factoring in both patient‐level consumption and system‐wide efficiencies.

This study describes a personal initiative by nephrology staff to deal with the water shortage. The information shared in this paper was collected based on direct interviews through asking open‐ended questions to the responsible nephrologists of Hassan II Dialysis Center in Oujda–Morocco using a structured questionnaire. The interviews were conducted and recorded by a professor of nephrology. The collected data was reorganized to ensure research quality. Our study adhered to the ethical principles of the Helsinki Declaration, and anonymity was not applicable.

A period of hydric stress is characterized by a significant reduction in water availability due to factors such as prolonged droughts, declining groundwater levels, or infrastructure limitations. In the case of our dialysis center, this refers to conditions where the municipal water supply is inconsistent, restricted, or at risk of depletion, necessitating proactive measures to ensure uninterrupted dialysis treatment.

## 3 Results

In response to periods of hydric stress, the Hassan II HD Center, a reference public HD center in eastern Morocco, has implemented several strategies to tackle water shortage and guarantee the uninterrupted functioning of HD procedures.

### 3.1 Increasing the Water Storage

One such measure involves the expansion of their water storage capacity: an additional tank has been installed in the treatment room, significantly increasing the center's water storage capacity from 2 tons to an impressive 5 tons. This enhancement represents a crucial step in safeguarding their water supply. The expansion of water storage capacity at the Hassan II HD Center has improved its resilience during water shortages. The previous 2‐ton capacity provided only 3.3 h of dialysis, whereas the new 5‐ton capacity extends emergency water reserves to 8.3 h. To maintain water quality, the center has implemented enhanced disinfection and monitoring measures, including chemical treatment (sodium hypochlorite or peracetic acid), UV sterilization, and regular testing of endotoxins, chlorine residuals, and bacterial counts. Additional strategies, such as temperature control and biofilm prevention, ensure water safety standards are upheld.

### 3.2 Improving the Water Distribution System

**FIGURE 1:**
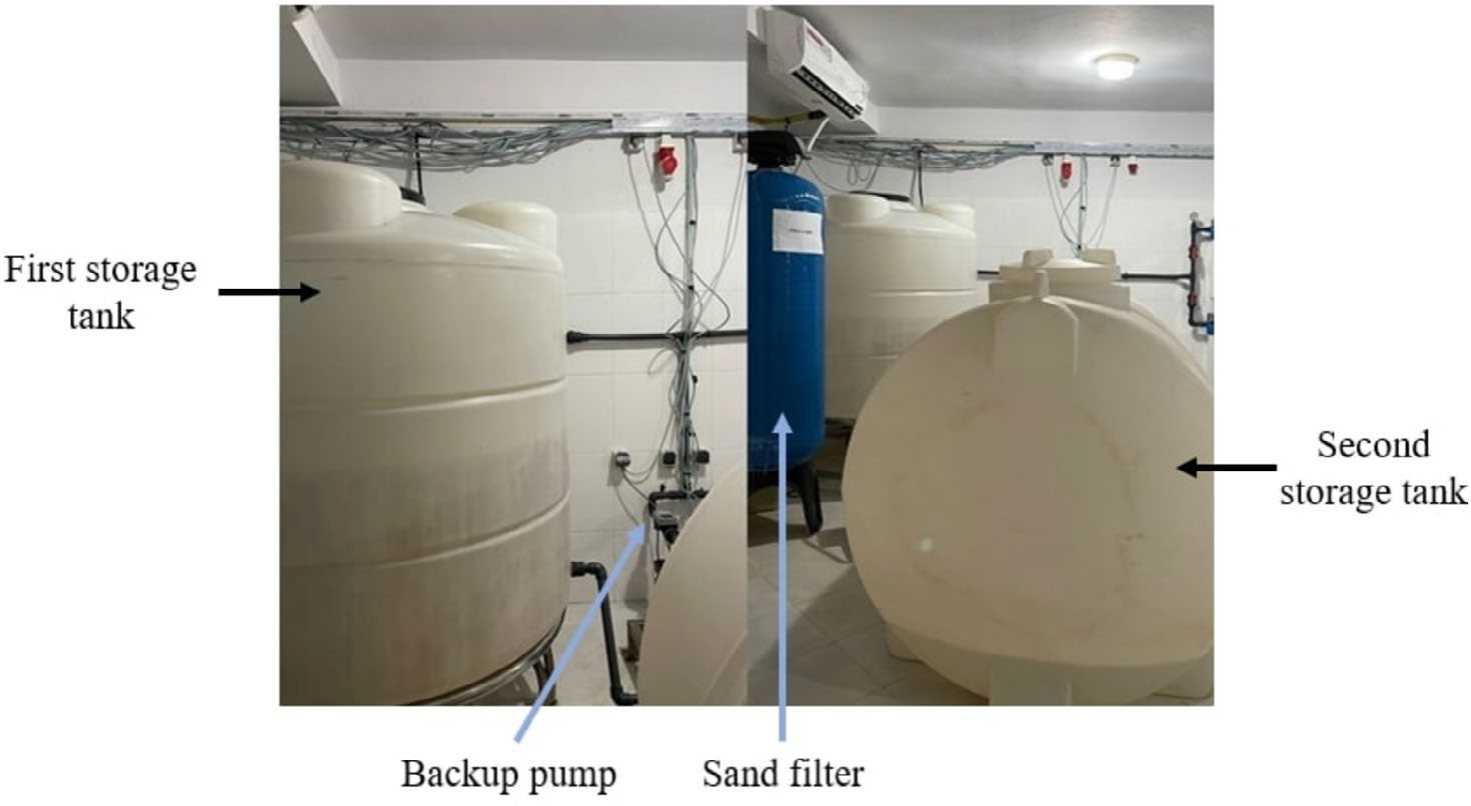
Additional tank to expand the water storage capacity.

In addition to increasing water storage, the center has taken proactive steps to fortify its internal water supply system (Figure 1). The initiative upgraded the Hassan II HD Center's internal water distribution system by replacing existing pipes with larger, more durable ones to reduce ruptures and ensure stable water flow. However, the municipal water supply network remained unchanged.

Given the high water demand of HD, the upgrade aimed to enhance resilience against pressure fluctuations, minimize maintenance disruptions, and improve efficiency. Although no formal cost–benefit analysis was conducted, similar investments have shown long‐term financial benefits by reducing water losses and maintenance costs.

As an outpatient dialysis center, the infrastructure reinforcement was tailored to its specific needs, unlike inpatient units requiring more extensive networks. These measures strengthen the facility's ability to provide reliable HD care despite water scarcity challenges.

### 3.3 Securing the Resource Monitoring

To enhance water security, the Hassan II HD Center has introduced a real‐time water level monitoring system with a sentinel alarm. This system detects critical reductions in stored water and promptly alerts staff, enabling timely intervention to prevent disruptions in dialysis procedures. By providing an early warning, it ensures a continuous and reliable water supply, which is essential for maintaining patient care.

When the system detects a drop in water levels below a predefined threshold, it immediately notifies specialized HD technicians. These technicians play a crucial role in assessing the severity of the situation, implementing emergency protocols, and optimizing water use. Additionally, they coordinate with nephrologists and nurses to make necessary adjustments to treatment plans, ensuring that patient safety and dialysis efficacy are maintained.

In cases where municipal water supply disruptions occur, technicians may activate emergency reserves to sustain ongoing dialysis sessions. To further extend water availability without compromising treatment quality, dialysate flow rates can be adjusted, and patient prioritization measures may be applied based on medical urgency.

If water shortages persist despite these interventions, additional measures may be required. These could include reducing flow rates within safe limits, slightly shortening session durations, rescheduling nonurgent treatments, or, in extreme cases, postponing some sessions to prioritize the most critical patients. While these strategies help mitigate the impact of water shortages, the potential for recurrent disruptions remains a significant challenge. Addressing this issue requires further research in nephrology literature, particularly in regions prone to water stress, to develop sustainable solutions for dialysis care under resource‐limited conditions.

### 3.4 Direct Communication With the Water Utility Provider

Recognizing the critical need for uninterrupted water access, the center has established an open line of communication with the local water supply authority, ensuring that water shortages do not disrupt dialysis services. This proactive engagement has allowed the center to raise awareness about the essential nature of HD and its high water dependency, receive early warnings about upcoming water restrictions or supply fluctuations, and advocate for prioritization of water delivery during municipal shortages to safeguard life‐sustaining treatments. While water shortages affect the broader community and region, this collaboration with local authorities has helped secure a more stable and predictable water supply for dialysis services. In cases where municipal resources are strained, water allocation plans have been adjusted to prioritize medical facilities, reducing the risk of abrupt treatment interruptions. However, dialysis prioritization remains a complex issue, especially in regions where severe water scarcity impacts multiple sectors, including households, agriculture, and industry. Although the center has benefited from a degree of preferential access, continued advocacy and integration into national emergency water management frameworks are essential to formalizing long‐term water security agreements for healthcare facilities.

### 3.5 Improving the Water Treatment

The Hassan II HD Center has improved its dialysis process by transitioning from a dual RO system to a more efficient single‐pass RO system. While the dual system ensured ultrapure water, it led to excessive water wastage. The single‐pass system, using only one filtration stage, significantly reduces water consumption while maintaining the required purity standards for HD.

This change has optimized water use by minimizing waste, simplifying operations, lowering maintenance and energy demands, and ensuring compliance with international dialysis water purity standards. It aligns with broader water conservation efforts, particularly in water‐scarce regions, without reducing patient sessions or compromising treatment quality.

Additionally, the HD center is exploring water regeneration strategies by assessing the feasibility of treating and reusing reject water. A comprehensive analysis of its physical, chemical, and microbiological properties is underway to ensure safe reuse. This initiative aims to establish a sustainable water source while maintaining stringent safety and quality standards for dialysis care.

## 4 Discussion

Our HD center sets a shining example of adaptation and sustainability in water management within a region increasingly impacted by water scarcity. Through a multi‐pronged approach—including strategic expansion of water storage, infrastructure upgrades, and cutting‐edge monitoring technology—we ensure continuous water availability for crucial HD procedures, even in times of extreme climate stress.

The growing urgency for health systems to adapt to climate change is widely recognized, yet preparedness remains inadequate across many regions. According to the WHO ATACH, healthcare systems must integrate climate resilience strategies to withstand environmental disruptions. However, as highlighted in the 2024 Lancet Countdown Report on Health and Climate Change, most health systems remain unprepared for the escalating effects of climate change, including water shortages, extreme heat, and climate‐sensitive infectious diseases [1, 2, 19]. Given the dependence of dialysis centers on water availability, this lack of preparedness poses a direct threat to kidney care systems, particularly in water‐stressed regions.

As water insecurity intensifies globally, kidney care administrators must integrate regional water security data, climate predictions, and expert insights into their planning. Their role in ensuring dialysis resilience is increasingly critical, requiring both daily operational strategies and disaster preparedness measures.

Unlike general conservation efforts, dialysis facilities need site‐specific, resilience‐focused water management strategies. Key measures include increasing on‐site water storage, integrating backup supplies, and using real‐time water quality monitoring. Infrastructure investments such as smart water metering, leak detection systems, and efficient purification processes help dialysis centers maintain operations despite municipal water fluctuations. Additionally, policies securing priority water access for medical use during droughts and emergencies should be promoted at regional and national levels [20].

Climate change is a major determinant of kidney health, reinforcing the importance of environmentally sustainable practices. The Green Nephrology concept highlights the significant resource demands of dialysis—high water, energy, and material consumption—along with substantial waste generation [21, 22, 23].

It is important to clearly distinguish between water scarcity adaptation and carbon footprint reduction in dialysis services. While both issues are critical to sustainable kidney care, water security efforts focus on ensuring consistent and sufficient water access for dialysis treatment, whereas carbon footprint reduction emphasizes minimizing emissions and energy consumption. These two concerns should be addressed separately to develop effective and targeted strategies [24].

Waste and resource management in dialysis impact both ecological sustainability and treatment costs, underscoring the need for integrated environmental and financial strategies [25].

Climate change has already contributed to reduced rainfall and groundwater depletion in many regions. Reports such as the IPCC Sixth Assessment confirm worsening water stress, while NASA GRACE data provide evidence of regional and global groundwater depletion [26, 27, 28]. Large‐scale infrastructure projects like desalination and water diversion require careful evaluation of long‐term feasibility, costs, and sustainability. Rather than relying solely on municipal expansions, dialysis centers should consider decentralized solutions, including rainwater harvesting, greywater reuse, and facility‐based recycling systems [29].

Anticipating potential crises is also crucial. If local water supplies run dry or neighboring regions face extreme shortages, leading to increased patient influx, contingency frameworks must account for emergency water agreements, facility scalability, and regional migration patterns [30]. Additionally, resilience planning should emphasize prevention by slowing the progression of chronic kidney disease and reducing acute kidney injury (AKI) through public health interventions, such as mitigating heat exposure, to ease the demand for dialysis services.

Expanding water supply infrastructure through projects like dam construction and desalination raises significant environmental concerns. Dams disrupt natural water cycles, alter sediment transport, and harm biodiversity, leading to ecological imbalances [31, 32]. They also have broader social, economic, and biological consequences [33]. Similarly, desalination, while providing an alternative water source, consumes large amounts of energy and discharges brine waste, which can harm marine life [34]. It can also lead to socio‐environmental issues such as public opposition and equity concerns [35]. Therefore, such “gray infrastructure” solutions must be assessed within a broader framework of sustainable water resource management, balancing their benefits against long‐term ecological costs.

As global water demand rises, societies must evaluate whether to prioritize increasing supply through large‐scale infrastructure or focus on reducing demand, improving efficiency, and enhancing conservation efforts. This question is particularly relevant to dialysis centers, which require substantial water resources while also facing broader water security and sustainability challenges [36, 37].

To address these concerns, kidney care providers must integrate climate‐informed water security measures into dialysis care, ensuring treatment continuity in water‐scarce regions. This requires cross‐sector collaboration, policy engagement, and the adoption of technologies that optimize water use [38].

Moreover, climate change exacerbates health risks linked to kidney disease. Rising temperatures increase insensible water and salt loss, heightening the risk of AKI and nephrolithiasis [39].

Simultaneously, climate change intensifies the frequency and severity of floods, which elevate the risk of diarrheal diseases and infections such as leptospirosis, hantavirus, malaria, and dengue—major contributors to AKI in low‐income and tropical regions [40]. Given these mounting threats, kidney care systems, particularly HD providers, must urgently develop adaptation frameworks to ensure resilience against water shortages and climate‐related health risks. To achieve long‐term water security, dialysis centers must go beyond basic conservation measures and integrate into regional and national water resilience frameworks. This includes securing priority water access agreements with local governments, investing in infrastructure to maintain operations during droughts, and adopting water‐efficient dialysis technologies. One of the most promising advancements is sorbent dialysis, which drastically reduces water consumption per session. Unlike conventional HD, which requires 120–160 L of water per treatment, sorbent dialysis uses only 5 L by continuously regenerating dialysate through a chemical sorbent system. This technology significantly reduces water waste and the logistical burden of water supply, making it particularly valuable for water‐scarce regions.

Additionally, operational adjustments such as reducing dialysate flow rates can help conserve water. Standard dialysis protocols typically use a dialysate flow rate of 500 mL/min, but studies suggest that lowering it to 300–400 mL/min could reduce water consumption while maintaining adequate solute removal. In our center, we use a dialysate flow rate of 375 mL/min for a blood flow rate between 250 and 300 mL/min, demonstrating a feasible reduction [41]. However, further research is needed to assess the long‐term impact of lower dialysate flow rates on dialysis adequacy and patient outcomes.

Despite its benefits, sorbent dialysis has not yet been widely adopted due to technological and commercial challenges. However, given the increasing urgency of climate adaptation and resource conservation, renewed interest in sorbent‐based systems may drive future innovation, particularly for home dialysis and resource‐limited regions. Future research should explore its feasibility, considering its potential to improve both environmental sustainability and healthcare resilience.

Ironically, healthcare itself contributes significantly to resource depletion and greenhouse gas emissions. Among medical therapies, dialysis has a particularly high environmental impact, leading to the emergence of green dialysis—a concept aimed at reducing the ecological footprint of dialysis treatment. Several countries have pioneered initiatives in this field, setting a baseline for sustainability and guiding future efforts [42, 43].

One key strategy involves reusing RO reject water. Agar et al. emphasize that this filtered water, which never comes into contact with patients, poses no infectious risk and should be incorporated into the general water use of dialysis facilities [44].

HD wastewater management presents another opportunity for sustainability. Studies have explored recycling dialysis effluent for agriculture, aquaponics, and industrial applications due to its nutrient‐rich content, including phosphate, urea, potassium, magnesium, and other bio‐useful substances. A Malaysian project demonstrated its potential use in aquaculture, hydroponics, and horticulture [45]. However, regulatory frameworks and cost–benefit analyses are needed to determine its feasibility. In France, Ponson et al. found that recycling dialysis effluent would require at least an ultrafiltration or additional RO system to mitigate infection risks, making the process economically unviable in the short to medium term [42].

**FIGURE 2:**
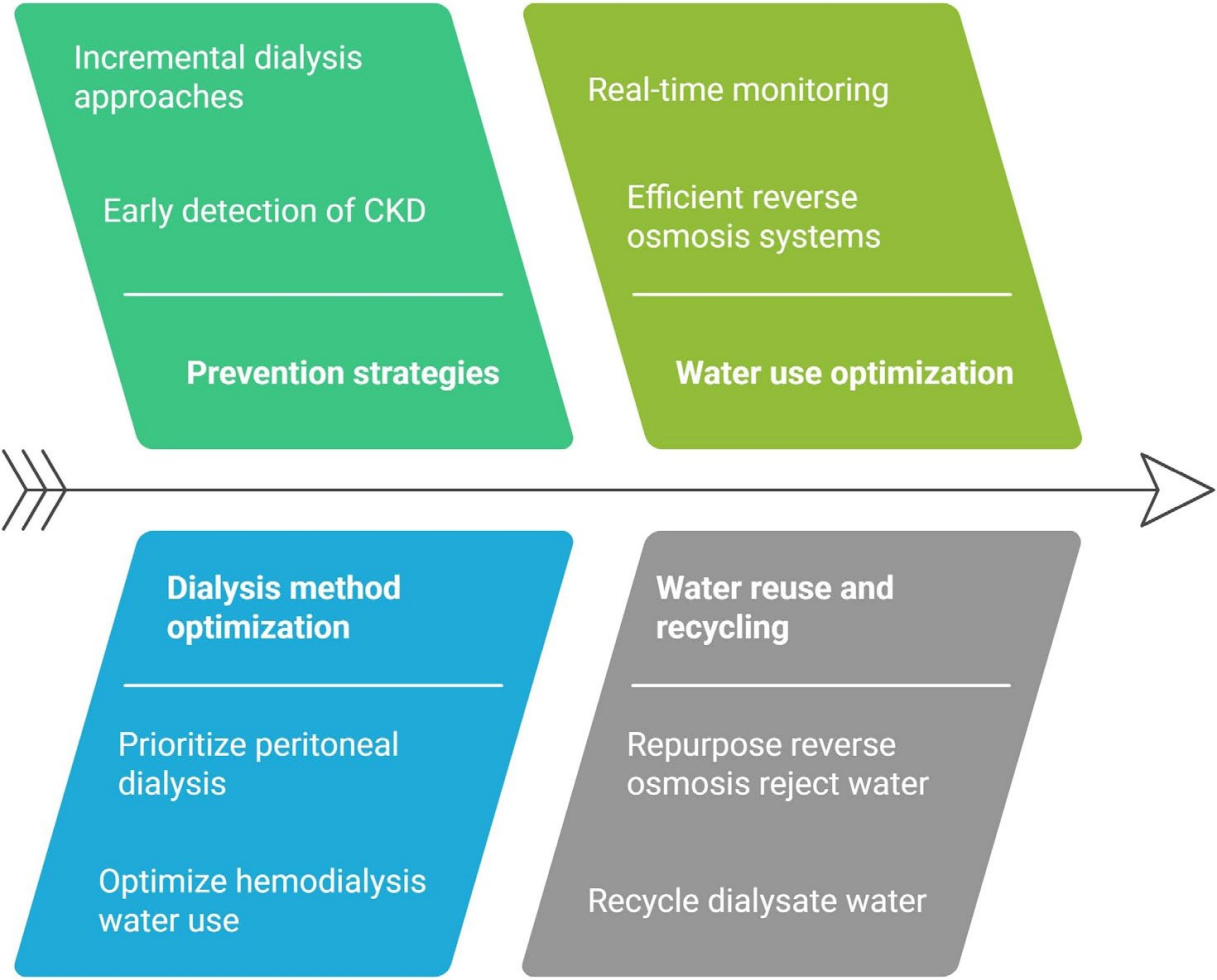
Adapting kidney replacement therapy to water scarcity.

Despite these challenges, innovative solutions continue to emerge. A Zero Liquid Discharge project implemented in a dialysis center in Casablanca, Morocco, by Tarrass et al. purifies and recycles HD wastewater, leaving little to no effluent, significantly reducing water waste and environmental impact [46]. Such initiatives highlight the potential for dialysis centers to adopt circular water management approaches, ensuring both economic and ecological sustainability in an era of increasing water scarcity (Figure 2).

Beyond water preservation, dialysis centers must also address their high carbon footprint. The Italian Society of Nephrology recommends several sustainability initiatives, including the use of renewable energy sources such as solar panels, energy conservation programs, and the integration of environmental sustainability into dialysis education. Additionally, encouraging eco‐friendly transportation options—such as public transport, cycling, and carpooling—can further reduce the environmental impact of HD [4].

Another major concern is the significant waste production in dialysis facilities. Materials that come into contact with body fluids are classified as potentially contaminated and require separate disposal. A single dialysis session generates between 1.5 and 8 kg of waste, depending on the waste management practices in place, particularly regarding the proper emptying of dialysis disposables [47]. Waste management begins with the materials used in packaging, often composed of plastic and paper. Effective triage and segregation of recyclable materials are essential to maximize reuse and minimize landfill contributions.

To enhance recycling efforts, staff training and collaboration between dialysis wards and local waste management facilities are crucial. Proper education on waste separation can significantly improve sustainability outcomes. The One Health philosophy reinforces the idea that human health reflects environmental health, emphasizing the need for hospitals and dialysis centers to serve as models of responsible resource use [48]. This principle should guide facility design, promoting the use of reusable materials (such as natural fiber bedding and lab coats), reducing reliance on single‐use synthetic consumables, and incorporating sustainable building materials whenever possible [4].

Finally, dialysis water security strategies must extend beyond individual centers to include regional and national policy advocacy. Governments and healthcare regulators should prioritize medical water access, integrate dialysis facilities into drought response plans, and incentivize the adoption of water‐efficient technologies. By combining institutional sustainability initiatives with broader policy engagement, dialysis providers can contribute to both environmental conservation and healthcare resilience in an era of increasing resource scarcity.

## 5 Conclusion

The rising demand for clean water clashes with its increasing scarcity, presenting a significant hurdle for healthcare, particularly in HD. This life‐saving treatment for kidney failure patients relies heavily on purified water, intensifying the pressure during water crises. However, amidst this challenge lie promising solutions that can ensure uninterrupted access to dialysis while promoting water conservation.

Innovative approaches to tackle water scarcity and HD are necessary, such as water conservation strategies (with the optimization of purification processes and HD machines), treatment protocol adjustments, and dialysate recycling. The promising future of alternative water sources usage and reuse of HD wastewater must be studied to lighten HD's heavy carbon footprint.

Addressing the water scarcity challenge in HD requires a collective effort. Healthcare providers, engineers, policymakers, and communities must work together to implement these solutions, considering the specific needs and contexts of different regions. A comprehensive approach should include prioritization of kidney transplantation and PD, developing robust contingency plans for water shortages, and advocating for public health measures to prevent kidney disease progression. By securing infrastructure investments and collaborating with water management authorities, dialysis centers can become truly sustainable in water‐stressed regions. Investing in research and development of water‐efficient technologies and exploring alternative water sources are crucial for long‐term sustainability. By embracing innovation and fostering collaboration, we can ensure that everyone, regardless of location or water scarcity, has access to the life‐saving treatment of HD.

## Conflicts of Interest

The authors declare no conflicts of interest.

## Data Availability

Data sharing is not applicable to this article as no new data were created or analyzed in this study.
